# Basal body multipotency and axonemal remodelling are two pathways to a 9+0 flagellum

**DOI:** 10.1038/ncomms9964

**Published:** 2015-12-15

**Authors:** R. J. Wheeler, E. Gluenz, K. Gull

**Affiliations:** 1 Sir William Dunn School of Pathology, University of Oxford, South Parks Road, Oxford OX1 3RE, UK

## Abstract

Eukaryotic cilia/flagella exhibit two characteristic ultrastructures reflecting two main functions; a 9+2 axoneme for motility and a 9+0 axoneme for sensation and signalling. Whether, and if so how, they interconvert is unclear. Here we analyse flagellum length, structure and molecular composition changes in the unicellular eukaryotic parasite *Leishmania* during the transformation of a life cycle stage with a 9+2 axoneme (the promastigote) to one with a 9+0 axoneme (the amastigote). We show 9+0 axonemes can be generated by two pathways: by *de novo* formation and by restructuring of existing 9+2 axonemes associated with decreased intraflagellar transport. Furthermore, pro-basal bodies formed under conditions conducive for 9+2 axoneme formation can form a 9+0 axoneme *de novo*. We conclude that pro-centrioles/pro-basal bodies are multipotent and not committed to form either a 9+2 or 9+0 axoneme. In an alternative pathway structures can also be removed from existing 9+2 axonemes to convert them to 9+0.

## Abstract (web summary)

Whether basal bodies are pre-committed to form 9+2 motile or 9+0 sensory axonemes and whether interconversion occurs between the two types of axonemes is not clear. Here, the authors used the unicellular eukaryote *Leishmania* as a model system to demonstrate that 9+0 axonemes can be formed *de novo* or by restructuring of 9+2 axonemes.

## Graphical abstract

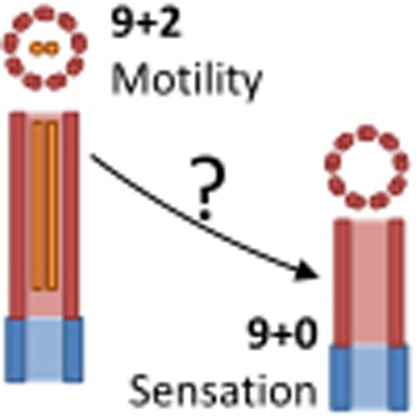

Eukaryotic cilia/flagella have two main characteristic ultrastructures for their two main classes of function; a 9+2 axoneme (nine outer microtubule doublets and a central microtubule pair) for motility, and a 9+0 axoneme (lacking the central pair) typical for sensory and signalling roles^1^,^2^. Ciliogenesis occurs by maturation of a centriole (termed a basal body when its primary function is nucleation of a cilium) that nucleates the ciliary axoneme, which then extends by addition of material to the distal end through intraflagellar transport (IFT)^3^,^4^. A cilium is made up of several hundred proteins^5^,^6^,^7^,^8^,^9^, with those involved in motility typically highly conserved and those involved in signalling or regulation of the flagellar beat having more species-specific adaptations. Major highly conserved structures (found across all eukaryotes) associated with the 9+2 axoneme are the inner and outer dynein arms (IDAs and ODAs), which generate the bending forces for movement^10^,^11^,^12^, the nexin links/dynein regulatory complexes (N/DRCs) that link the outer doublets^13^,^14^, and the radial spoke complexes (RSCs) and central pair projections (CPPs) that coordinate the flagellar beat^15^,^16^,^17^,^18^. In a 9+2 axoneme, the central pair is nucleated at the basal plate at the end of the transition zone, and it is at this point that incorporation of IDAs, ODAs, N/DRCs, RSCs and CPPs start; however, when and how a 9+2 or 9+0 axoneme structure is defined is not well understood.

Whether transitions can be made between structures and functions of cilia post-assembly is not clear, despite ciliary structure and functions being extremely highly conserved: the last common eukaryotic ancestor could assemble a motile cilium and cilia, and centrioles are present in all major branches of extant eukaryotic life^19^,^20^. Examples of species that form cilia both with and without a central pair for different functions are known in the chromalveolata (*Dileptus*^21^), excavata (*Leishmania*^22^) and holozoa (with many examples, particularly in metazoa including humans and *Drosophila*^1^). In humans, defects in either sensory or motile cilia can cause severe disease showing correct assembly and function of cilia is critical^23^,^24^. The ciliopathies (for example, Kartagener's, Meckel-Gruber, Bardet-Biedl and Alström syndrome) constitute a diverse set of syndromes exhibiting phenotypes such as chronic lung problems, male and female infertility and developmental defects^23^,^25^, congenital retinal blindness and polycystic kidney disorders^24^,^26^,^27^,^28^. The phenotypes of individual ciliopathies reflect the diverse but essential roles played by motility and sensory molecules in different cilia in different tissues. Generating a cilium with the correct structure necessary for its function is a cellular ability shared across much eukaryotic life, and is critical in humans. How and when such differing ciliary forms are specified and whether they are interchangeable after formation are important open questions for developmental cell biology.

Cilia and flagella form by extension from centrioles and basal bodies. Whether centrioles/basal bodies are pre-committed to one type of cilium or whether they are ‘multipotent' is not understood. Much is known about the dual function of centrioles/basal bodies in the interphase and mitotic cell and during the pathways of cilia formation; new centrioles are typically nucleated to the side of an existing ‘mother' centriole with cell cycle-regulated serine/threonine kinases (including Aurora and Polo-like kinases) triggering centriole assembly^3^, which starts by the generation of nine-fold symmetry by the assembly of a SAS-6 cartwheel, to which the microtubules and other proteins are added^29^. The new centriole typically remains immature for one cell cycle, before it can be used as a basal body or to nucleate a new daughter centriole^3^. Surprisingly no evidence exists on whether a basal body subtending a 9+2 axoneme can be reused to subtend an axoneme without a central pair, or *vice versa*. The cell developmental pathways available for ciliary axoneme structure conversion and whether there is commitment of a basal body to nucleate a particular axoneme structure has profound implications for cell development and differentiation.

The unicellular eukaryotic parasite *Leishmania mexicana* offers an opportunity to address these questions. *L. mexicana* can form a long motile flagellum with a canonical 9+2 axoneme, similar to both humans and *Chlamydomonas*, and possesses homologues of many proteins found in the molecular machinery of the *Chlamydomonas reinhardtii* and human 9+2 axoneme (Table 1, Supplementary Data 1). *L. mexicana* can also form a shorter immotile flagellum with a 9v axoneme (a 9+0 axoneme that has a collapsed radial symmetry with irregular inward migration of the outer doublets). This 9v organization mirrors precisely the structure found in the mammalian primary cilium^22^. The promastigote life cycle stage (found in the sandfly vector) has the 9+2 axoneme^30^,^31^, while the amastigote life cycle stage (found inside macrophages in the mammalian host) has the 9v axoneme^22^ (Fig. 1a).

We analysed *L. mexicana* flagellum length, structure and biochemical changes, using electron microscopy and cell lines-expressing enhanced green fluorescent protein (eGFP) fusions of axonemal proteins, through the promastigote to amastigote life cycle transition. This shows that (1) 9+0 axonemes can form by both *de novo* growth and by restructuring of existing 9+2 axonemes through axoneme disassembly associated with decreased IFT and (2) pro-basal bodies formed under conditions where a 9+2 axoneme would normally form can switch to form a 9+0 axoneme. We conclude that immature pro-basal bodies are ‘bipotent' and are not committed to form either a 9+2 or 9+0 axoneme and 9+0 axonemes can be also formed by restructuring of existing 9+2 axonemes.

## Results

### Tools for analysing axoneme structure change

**Figure 1:**
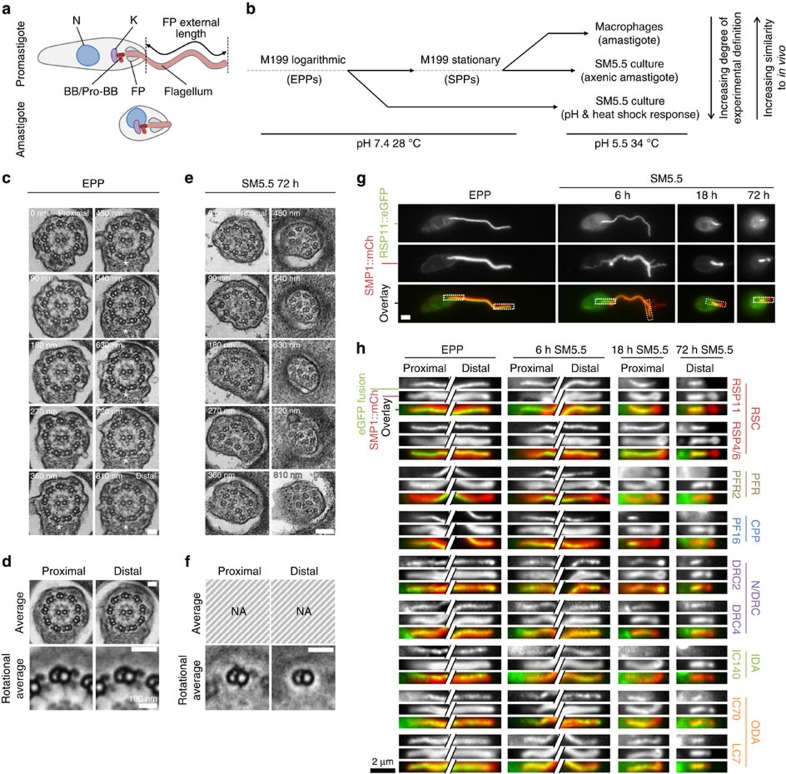
The *Leishmania mexicana* promastigote and amastigote and their flagellar axoneme structures. (**a**) Cartoons of promastigote and amastigote morphology. The nucleus (N), kinetoplast (K), basal body (BB) and pro-BB, flagellar pocket (FP) and FP-external flagellum length (used for length measurement) are shown. (**b**) The three *in vitro* models of life cycle differentiation used. (**c**) TEMs of serial sections through the FP of a single EPP showing a 9+2 axoneme structure and the start of the PFR in distal sections (asterisks). (**d**) Average axoneme electron density of the proximal five and distal five sections, and outer doublets after ninefold Markham rotational averaging. CPPs, IDAs, ODAs, N/DRCs and RSCs are visible. (**c**,**d**) Representative of five cells. (**e**) TEMs of serial sections through the FP of a single parasite 72 h after transfer of EPPs to SM5.5 showing lack of a central pair and irregular collapse of the outer doublets (9v structure). (**f**) Outer doublets after ninefold Markham rotational averaging from the proximal five and distal five sections. Only ODAs and RSCs are visible proximally, no doublet decorations are visible distally. (**e**,**f**) Representative of 10 cells. Scale bars in **c**–**e** represent 50 nm. (**g**) Example fluorescence micrographs of an EPP and parasites 6, 18 and 72 h after transfer of EPPs to SM5.5. This example cell expressed RSP11::eGFP (an axonemal protein) and SMP1::mCh (a flagellar membrane marker). (**h**) Summary of fluorescence in EPPs and cells 6, 18 and 72 h after transfer of EPPs to SM5.5 from panel 1 and panel 2 of cell lines expressing eGFP fusions of known axonemal proteins (Table 1). Crops of the proximal and distal flagellum (EPPs and 6 h) or the whole flagellum (18 and 72 h) are shown, as indicated in **g**. All proteins localized along the entire EPP flagellum (except PFR2::eGFP which was present from mid-way through the flagellar pocket). By 72 h the PF16::eGFP, PFR2::eGFP and IC140::eGFP signal was absent from flagella and signal from other proteins was present in the proximal half of flagella. SMP1::mCh gained a constriction in width near the flagellar pocket exit. Scale bars in **g**,**h** represent 2 μm.

We used three *in vitro* models of *L. mexicana* life cycle stage differentiation to analyse flagellum length and structure change during the promastigote to amastigote transition (Fig. 1b): (i) entry of stationary phase promastigotes (SPPs) into macrophages to generate amastigotes, (ii) mimicking the conditions of the macrophage phagolysosome by transfer of SPPs into SM5.5 medium (34 °C and pH5.5) to generate axenic amastigotes^32^ and (iii) transfer of exponentially growing promastigotes (EPPs) to SM5.5. SPPs are enriched in cells morphologically similar to the transmissive metacyclic promastigote forms found in the sandfly mouth parts^33^,^34^,^35^ (Supplementary Fig. 1a–c). The first two models are well established^32^,^36^,^37^ and generate an amastigote cell with a short 9v axoneme, and we show here that the third model was also sufficient to generate cells with amastigote morphology and a short 9v axoneme in 72 h, assessed by transmission electron microscopy (TEM; Fig. 1c–f). We also generated a set of cell lines expressing eGFP fusions of axonemal proteins^13^,^15^,^38^,^39^,^40^,^41^,^42^ (Table 1, Supplementary Fig. 2a-b), which showed changes to axoneme molecular composition after transfer of EPPs to SM5.5 consistent with TEM (Fig. 1g,h) and similar to those during SPP infection of a macrophage (Supplementary Fig. 2c).

During the *Leishmania* cell cycle, a new flagellum is produced and the old and new flagellum are partitioned to two separate daughters^43^. Assessment of how the short amastigote 9v flagellum is formed must consider whether a pre-existing promastigote 9+2 axoneme can restructure to 9v, whether the 9v axoneme is assembled in a new flagellum during a differentiation division, or a combination of both. To this end it was important to assess changes in flagellar length and occurrence of division during differentiation in all three models.

### Existing flagella shorten and new short flagella form

**Figure 2:**
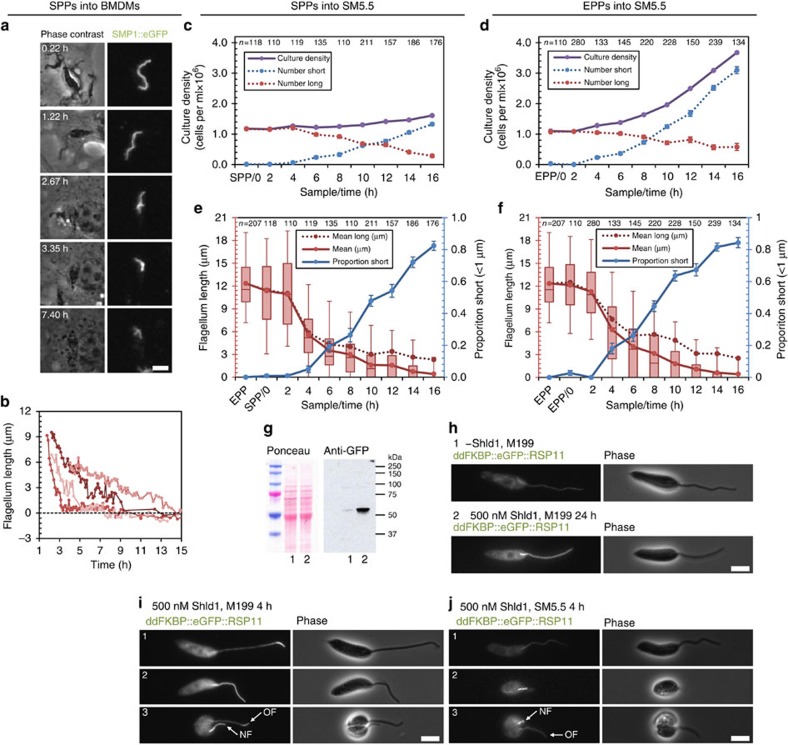
Promastigote to amastigote differentiation involves flagellum shortening and can include division events. (**a**) Frames from Supplementary Movie 1 illustrating flagellum length shortening following uptake of SPPs expressing SMP1::eGFP by a macrophage. (**b**) Flagellum length of four parasites following uptake by a macrophage, representative (*n=*38) of the range of flagellum shortening rates. (**c**,**d**) Culture density following transfer of (**c**) SPPs or (**d**) EPPs to SM5.5. Number of cells with short or long flagella (<1 μm or >1 μm) were calculated from the proportion observed by light microscopy (**e**,**f**). Error bars indicate standard error of proportion (s.e.p.). (**e**,**f**) Mean flagellum length (red), proportion of cells with a short flagellum (blue) and mean flagellum length of only cells with a long flagellum (dotted red) following transfer of (**e**) SPPs or (**f**) EPPs to SM5.5. Error bars indicate s.e.p. Box and whiskers represent median, upper and lower quartiles and 5th and 95th percentiles. Data in **c**–**f** are representative of two independent experiments. (**g**) Anti-GFP western blot of lysate from EPPs expressing ddFKBP::eGFP::RSP11 with 500 nM Shld1 for 24 h (lane 2) or without Shld1 (lane 1). ddFKBP::eGFP::RSP11 is unstable in the absence of Shld1. (**h**) Representative fluorescence micrographs of live cells from the samples in **g** demonstrating induction of flagellum labelling by addition of Shld1. (**i,j**) Fluorescence micrographs of (**i**) EPPs 4 h after addition of Shld1 and (**j**) 4 h after transfer of EPPs to SM5.5 with Shld1. Scale bars in **a**,**h**–**j** represent 5 μm.

Time-lapse microscopy of SPPs (with long 9+2 flagella) entering macrophages (Supplementary Movie 1) showed 87.3% were viable, with the parasite membrane remaining intact and impermeable to rhodamine–dextran for 16 h after uptake. These parasites expressed SMP1::eGFP, a *Leishmania-*specific small myristoylated protein that is present at a high concentration on the inner face of the flagellar membrane^44^, as a bright fluorescent flagellum marker independent of internal flagellum structure. For viable cells, the long 9+2 flagellum shortened over the first 16 h with no division (*n*=102) until no flagellum extended beyond the flagellar pocket (Fig. 2a). Shortening rates varied widely and followed decelerating kinetics (Fig. 2b) with flagella, on average, reducing in length by a factor of 0.265 each hour (a median shortening rate constant of 0.265 h^-1^ (*n*=38)). Shortening rate showing no correlation with starting flagellum length (Pearson's *R*^*2*^=0.20). In both axenic differentiation models both flagellar shortening and cell division occurred. Following transfer of SPPs to SM5.5 population growth was limited (Fig. 2c), while following transfer of EPPs to SM5.5 the population grew with a doubling time of ∼8 h (Fig. 2d). In both models, the proportion of cells with a short amastigote-like flagellum (<1 μm flagellar pocket-external length) increased, more than accounting for population growth (Fig. 2c–f). Mean flagellum length excluding these short flagella also decreased (Fig. 2e,f). Together, this initial data suggested the possibility that both new flagellum formation and flagellum shortening may contribute to generating the 9v amastigote axoneme.

Promastigote flagella are normally in a growing state^43^, so the shortening of flagella in all three differentiation models appears to be a switch to a disassembling state. We confirmed this using a cell line expressing ddFKBP::eGFP::RSP11. The N-terminal ddFKBP domain causes proteasome-mediated degradation of the protein in the absence of the stabilizing ligand Shld1 (ref. 45) (Fig. 2g,h). A short incubation with Shld1 can, therefore, be used to label newly incorporated ddFKBP::eGFP::RSP11 into a flagellum. Incubation of EPPs with Shld1 for 4 h showed 96% (*n*=111) of cells incorporated ddFKBP::eGFP::RSP11, either at just the flagellar tip (60%; Fig. 2i top panel), showing flagellum growth and/or material turnover, or along the entire flagellum length (36%; Fig. 2i middle panel), indicating a newly grown flagellum. Dividing EPPs with two flagella incorporated ddFKBP::eGFP::RSP11 at the tip of the old flagellum and along the entire new flagellum (Fig. 2i; bottom panel). This showed that all EPP flagella are growing, or at least undergoing turnover of material at the flagellum tip similar to *Chlamydomonas*^46^. In contrast, if the EPPs were transferred to SM5.5 before Shld1 addition, 93% (*n=*67; Fig. 2j top panel) of cells with a long flagellum had incorporated no ddFKBP::eGFP::RSP11, showing no flagellum growth or turnover. All flagella that had incorporated ddFKBP::eGFP::RSP11 along their entire length were very short (Fig. 2j; middle panel). Dividing cells 4 h after transfer to SM5.5 only had signal along the length of the new flagellum (Fig. 2j; bottom panel). This confirmed that flagellum shortening is a switch from a growing to disassembling state (which may be associated with a cessation of turnover of flagellar material), and shortening contributes to forming the short amastigote flagellum.

**Figure 3:**
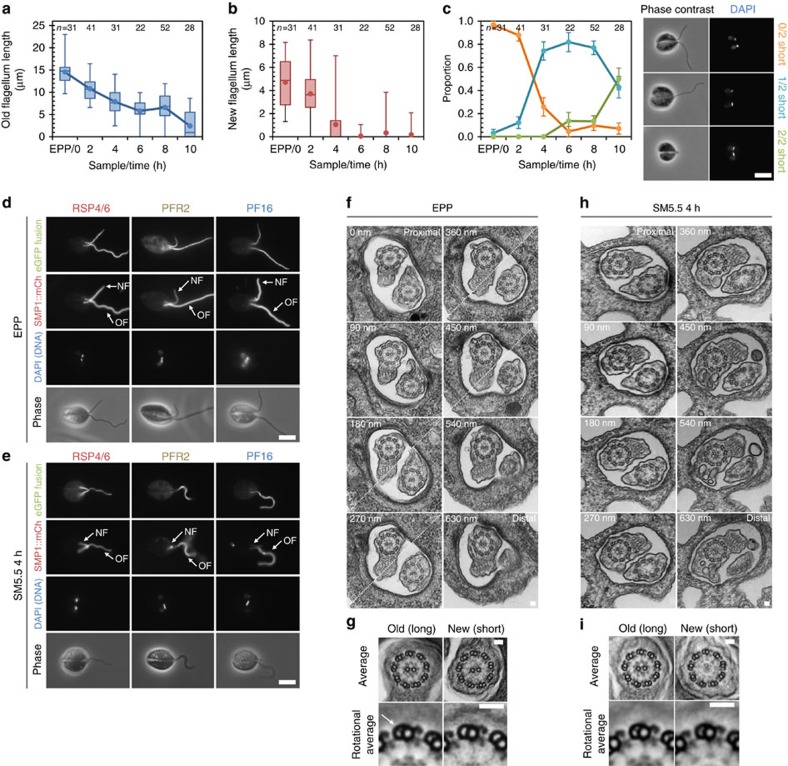
Axonemes formed *de novo* are 9+0 and grow next to an existing 9+2 flagellum. (**a**) Longer (older) flagellum length on dividing (1K2N and 2K2N) cells following transfer of EPPs to SM5.5. (**b**) Shorter (newer) flagellum length on dividing cells following transfer of EPPs to SM5.5. Points represent the mean and box, and whiskers represent median, quartiles and 5th and 95th percentiles in **a**,**b**. (**c**) Proportion of dividing cells with zero, one or two short flagella (<1 μm) following transfer of EPPs to SM5.5. Error bars indicate standard error of proportion. Example phase contrast and DAPI fluorescence micrographs of the three classes of dividing cell are shown. Data in **a**–**c** are representative of two independent experiments. (**d**,**e**) Native fluorescence micrographs of dividing cells expressing eGFP fusions of known axonemal proteins from our first panel of cell lines; (**d**) as EPPs with zero short flagella and (**e**) cells with one short flagellum 4 h after transfer to SM5.5. Axoneme composition was identical in both EPP flagella; 4 h after transfer to SM5.5, PFR2::eGFP and PF16::eGFP did not localize to the short, new flagellum (NF). Scale bars in **c–e** represent 5 μm. (**f**) TEMs of serial sections through the two flagella in the flagellar pocket (FP) of a single dividing EPP. Diagonal dashed lines indicate that the image is a composite of two different sample tilt angles. (**g**) Axoneme averages and detail of doublets after Markham rotational averaging of the old and new flagellum. The only structural difference between the new and old axoneme was the absence of ponticuli (arrow) from the lumen of the B-tubule of outer doublets in the new flagellum. (**h**) TEMs of serial sections through the two flagella in the FP of a single dividing cell 4 h after transfer of EPPs to SM5.5. The central pair is absent from the new flagellum. (**i**) Axoneme averages and detail of doublets after Markham rotational averaging of the old and new flagellum. The only structural differences are the absence of ponticuli and central pair in the new flagellum. **f**,**g** and **h**,**i** are each representative of five cells. Scale bars in **f**–**i** represent 50 nm.

To address the contribution of new flagellum formation, we first asked whether dividing cells possessed a short amastigote-like newly formed flagellum and a long flagellum after transfer of EPPs to SM5.5 (where division was common (Fig. 2d)). We measured flagellum length on individual dividing cells at two hourly intervals for 10 h following transfer of EPPs to SM5.5 (Fig. 3a-c). The average length of the old (longer^43^) flagellum gradually decreased from ∼15 μm to ∼1 μm (Fig. 3a). The length of newly formed (shorter) flagellum showed a sharp switch from reaching ∼4 μm to <1 μm, barely protruding from the flagellar pocket, in division events 4 h after transfer to SM5.5 (Fig. 3b). We categorized these biflagellate dividing cells as having zero, one or two short amastigote-like flagella (Fig. 3c). The proportion with zero short flagella (similar to normal promastigote division^43^) decreased rapidly after 2 h, while the majority of early division events (4 to 8 h) were asymmetric, with one short flagellum. By 10 h, division events with two short flagella (similar to normal amastigote division) were in the majority. This showed that when cells with an existing long flagellum divided for the first time in SM5.5, a new short amastigote-like flagellum grew alongside the pre-existing long flagellum.

### Newly formed axonemes are 9+0

To test whether new short flagella formed during division events in SM5.5 were 9+0, we used panel 1 of cell lines expressing eGFP fusions of known axonemal proteins and TEM to examine the structure of the old and new flagellum in dividing EPPs before and 4 h (approximately half of one cell cycle period (Fig. 2d)) after transfer to SM5.5. In EPPs, RSP4/6::eGFP, PFR2::eGFP and PF16::eGFP localized along the entire length of both flagella of dividing cells (Fig. 3d). In cells that were in division 4 h after transfer to SM5.5, only RSP4/6::eGFP was detected in short new flagella. PFR2::eGFP and PF16::eGFP were absent (Fig. 3e) suggesting an absence of the central pair and paraflagellar rod (PFR). We confirmed this result using TEM of the flagellar pocket region of dividing parasites. Old flagella in kinetoplastid parasites can be recognized by intra-lumenal projections (ponticuli) in the B-tubule of the outer axonemal doublets^47^. Therefore, the new flagellum in dividing cells could be identified by the absence of ponticuli. In dividing EPPs before transfer to SM5.5 both old and new axonemes were 9+2 (Fig. 3f), and averaging and Markham rotation averaging of the axoneme TEMs showed both had CPPs, RSCs, N/DRCs, IDAs and ODAs (Fig. 3g). Four hours after transfer of EPPs to SM5.5 the old axoneme was 9+2, but the new axoneme was 9+0 (Fig. 3h) and lacked CPPs, but still had RSCs, N/DRCs, IDAs and ODAs (Fig. 3i). Importantly, this is not a collapsed 9v structure, which is presumably acquired later; see below. This shows that when exposed to differentiation conditions, a *L. mexicana* pro-basal body can mature and nucleate a new 9+0 flagellum alongside an existing 9+2 flagellum.

This *Leishmania* system allows us to address whether a basal body is pre-committed to forming a 9+2 or 9+0 axoneme or is multipotent. Our above choice of 4 h post transfer to SM5.5 for the analysis of dividing cells is significant; given the 8 h doubling time of these EPPs after transfer to SM5.5, we can know that the basal body from which the new flagellum was nucleated had to be formed in the previous proliferative promastigote cell cycle^43^ where the flagella were 9+2 and a newly formed flagellum would be 9+2. Therefore, we can conclude that a newly formed pro-basal body/pro-centriole is multipotent and only acquires the determinants for a 9+2/9+0 pathway during the maturation process.

### Existing 9+2 axonemes can restructure to 9+0

**Figure 4:**
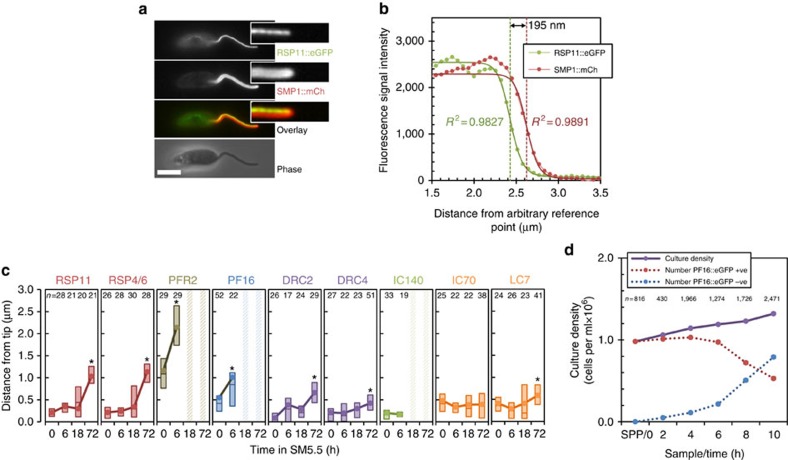
9+2 axonemes can disassemble the central pair and so restructure to a 9+0 axoneme. (**a**) Axoneme tip structure was super-resolved by analysing the signal intensity profile across the flagellum tip. Example native fluorescence micrograph of a live cell from M199 expressing RSP11::eGFP and SMP1::mCh, with insets of cropped and digitally straightened flagellar tips. Scale bar represents 5 μm. (**b**) Signal intensity profiles of eGFP and mCh fluorescence signal from the straightened flagellar tip in **a** fitted to a modified Rodbard four-parameter logistic curve. The distance between half maximum signal was taken as the distance between the tip of the eGFP and mCh containing structures. (**c**) Axoneme tip structures of EPPs and cells 6 , 18 or 72 h after transfer of EPPs to SM5.5, determined using the methods in **a**,**b** in our first and second panel of cell lines (Table 1). Data points indicate the mean, boxes represent the median and upper and lower quartiles. Time points where that axoneme component was absent in the majority (>50%) of cells are indicated with a hatched bar. Significant changes from 0 h are indicated with an asterisk (Welch's *t*-test, *P<0.01). (**d**) Culture density measured at 2-h intervals for 10 h following transfer of SPPs expressing PF16::eGFP to SM5.5. Proportion of cells retaining PF16::eGFP signal anywhere in the flagellum were counted from epifluorescence micrographs and used to calculate the number of cells retaining PF16::eGFP. The number of cells with PF16::eGFP signal decreased. Error bars indicate standard error of proportion. Representative of two independent experiments.

Having established the division-dependent method of acquisition of an altered flagellum form, we now returned to question whether an existing 9+2 flagellum can restructure to a new form. In the *Leishmania* system 9+2 flagella can shorten to an amastigote-like length (Fig. 2a–f). However, this does not show whether a 9+0 form or molecular composition has been reached. The localization of eGFP-tagged axonemal components showed that the 9v axoneme lacked the PFR, CPPs and IDAs. They also lacked RSCs, ODAs and N/DRCs in the distal half (Fig. 1e,f,h). This is the same as in parasites differentiating *in vivo* during infection of macrophages (Supplementary Fig. 2c) and unlike the promastigote axoneme where all these structures are present (Fig. 1c,d,h). Given these patterns, we examined the flagellum tip organization of differentiating parasites following transfer of EPPs to SM5.5, to test for changes indicative of restructuring associated with disassembly. For reference, we first examined the structure of the flagellum tip in EPPs with growing 9+2 flagella. The distance between the distal end of the structure containing the eGFP-tagged axoneme protein and the membrane bounding the flagellum tip (marked with SMP1::mCh) was determined from signal intensity profiles (Fig. 4a,b). This method is based on determining the relative location of flagellum structure tips in two channels, rather than resolving structures, allowing measurement of flagellum tip structure at greater than the resolution of the light microscope. RSC, IDA and N/DRC components in EPPs were present to within 0.2 μm of the flagellum tip (Fig. 4c). These structures were the closest to the flagellum tip, suggesting they were assembled first in growing 9+2 axonemes. ODA and CPP components lagged in incorporation by 0.2 μm, and PFR lagged by a further 0.6 μm (Fig. 4c), indicating later assembly. This pattern of RSC, CPP and PFR assembly was the same as that described for growing 9+2 flagellum tips in *Trypanosoma brucei*^48^. We then examined the structure of the flagellum tip 6 h after EPPs were transferred to SM5.5, by which time the axonemes have changed to a disassembling state (Fig. 2g–j). Tip structure was the same as during growth except for CPP and PFR components, which were further from the flagellum tip (Fig. 4c). This showed the PFR, CPPs and the central pair (as PF16 is required for its stability^38^) are the first structures lost from disassembling 9+2 axonemes, and can be removed ahead of the outer doublet-associated structures.

If true, this would predict that in differentiation from SPPs, where there is limited division and formation of new flagella, a large proportion of cells with no central pair could arise from axoneme restructuring. We analysed SPPs expressing PF16::eGFP at two hourly intervals for 10 h following transfer to SM5.5. The number of cells retaining PF16::eGFP signal dropped rapidly, at a rate too fast to be accounted for by new flagella from division events (Fig. 4d). Shortening flagella were, therefore, completely losing PF16::eGFP. Since PF16::eGFP was expressed with a 3′ untranslated region (UTR) that drives constitutive expression throughout the life cycle, this indicated loss of the structure to which PF16::eGFP binds (the central pair) rather than downregulation of PF16::eGFP expression. We, therefore, conclude that complete loss of CPPs and the central pair by the disassembly from a 9+2 axoneme is possible.

Generation of the 9v amastigote axoneme from an intermediate 9+0 axoneme requires distal loss of RSCs, ODAs and N/DRCs (Fig. 1e,f,h). To determine whether and when this occurs, we looked at later time points after transfer of EPPs to SM5.5. By 18 h, the majority of flagella lacked PFR, IDAs and CPPs, but still had RSCs, ODAs and N/DRCs all the way to the distal tip (Figs 1e,f,h and 4c). This population is a mixture of both *de novo* generated 9+0 axonemes, and those generated by 9+2 flagellum shortening and central pair loss. There is a further major reduction in radial spoke and other components by 72 h, indicative of the length of time needed to acquire the 9v axoneme.

### Flagellum restructuring is associated with decreased IFT

**Figure 5:**
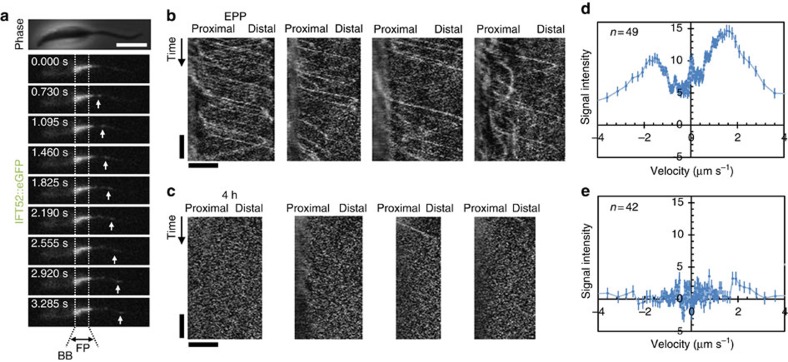
9+2 axoneme shortening and restructuring to 9v is associated with a reduction in IFT. (**a**) Frames from Supplementary Movie 2 illustrating IFT52::eGFP localisation and movement in IFT particles in EPPs. The approximate position of the basal body (BB) and flagellar pocket (FP) are indicated. (**b**,**c**) IFT activity in EPPs, shown by (**b**) representative kymographs of IFT52::eGFP movement and (**c**) quantitation of fluorescence intensity moving with an anterograde (positive) or retrograde (negative) velocity. (**d,e**) IFT activity 4 h after transfer of EPPs to SM5.5, shown by (**d**) representative kymographs and (**e**) quantitation of fluorescence. Error bars indicate s.e. Horizontal scale bars represent 5 μm, vertical scale bars represent 5 s.

Given this remodelling of the flagellum, we asked whether we could observe any associated changes in intrinsic flagellar biochemistry that could suggest a mechanism. First, we asked whether one could detect concomitant changes in the occurrence of the few known flagellar tip proteins^49^,^50^. Kin13-2::eGFP and eYFP::CEP104 localized to the tip of growing 9+2, disassembling 9+2, 9+0 and 9v axonemes (Supplementary Fig. 3), indicating no modulation during the restructuring processes and suggesting that they associate with the doublet tips or membrane rather than the central pair. Second, we asked whether we could detect any change in the pattern of IFT to and from the flagellum tip. We tracked IFT using a cell line expressing IFT52::eGFP, a member of IFT complex B^51^. In EPPs IFT52::eGFP localized to the flagellum, with a high signal around the basal body/transition zone region and with moving particles along the flagellum (Fig. 5a, Supplementary Movie 2), similar to the pattern seen in *Trypanosoma brucei*^52^. We generated kymographs from EPP flagella that, although noisy, showed both anterograde and retrograde particles, typically travelling at similar speeds, with occasional slower anterograde particles (Fig. 5b). We quantified image intensity corresponding to tracks of different speeds from the fast Fourier transforms (FFTs) of kymographs. The typical speed of anterograde particles was 1.6 μm s^-1^ (similar to *T. brucei*^52^ and *C. reinhardtii*^53^) and that of retrograde particles was 1.5 μm s^-1^ (slower than *T. brucei* or *C. reinhardtii*^52^,^53^; Fig. 5c). Four hours after transfer of EPPs to SM5.5 long flagella, which have now switched to a state of disassembly, showed a massive reduction in number of IFT particles (Fig. 5d,e). This indicated that flagellum disassembly and restructuring is associated with a global reduction in transport potential within the flagellum.

## Discussion

Within particular constraints there is an intriguing structural and functional diversity in the ciliary/flagellar axoneme throughout evolution^20^. Diversity is also seen at the level of single organisms, with adaptations of flagellum structure and function specific to different tissues or in different stages of a life cycle^1^,^20^. The capacity of an organism to form both a 9+2 motile cilium and a 9+0 sensory cilium is presumably the most common example. *Leishmania* is an excellent experimental model with which to assess how such transitions can occur and the level of control over them, as it is an example where a single cell can switch from building a 9+2 cilium to a 9+0 cilium. Furthermore, this transition can be triggered *in vitro*, with the simple environmental cues of pH and temperature^37^, and efficient homologous recombination of transfected DNA allows easy generation of transgenic *Leishmania* cell lines^54^ for the dissection of the underlying molecular mechanisms.

**Figure 6:**
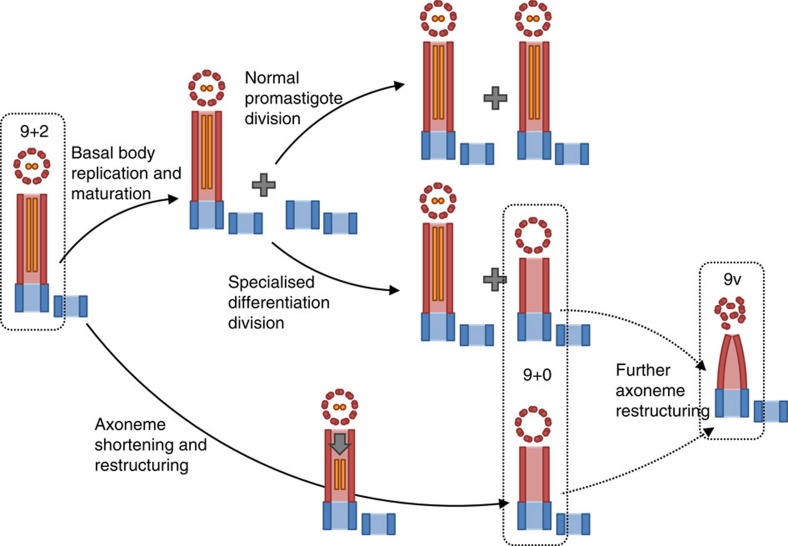
Cartoon summarising the pathways for 9+2 to 9v axoneme conversion in *Leishmania*. The basal body and pro-basal body are shown in blue, the outer doublets in red and the central pair in orange. The major pathway in macrophages is likely to be axoneme shortening and restructuring.

Our results show that a pre-existing motile cilium has a level of plasticity allowing it to be remodelled over a period of time, in this case allowing it to adapt to the structure necessary for an intracellular life style as a pathogen within mammalian macrophages. In this system, this included preferential disassembly of the central pair, causing conversion of a 9+2 axoneme to 9+0 (Fig. 6). This flexibility may mean that axoneme structure should be considered as a continuum of possibilities, rather than strict classes. There are several mechanisms by which 9+2 to 9+0 axoneme structure change could be triggered, including loss of a stabilizing cap structure from the axoneme tip, destablization of the constituent microtubules of the axoneme or through regulation of IFT or the cargo it delivers to the flagellum tip. We saw no change in the localization of the axoneme tip proteins CEP104 and Kin13-2 during flagellum shortening and loss of the central pair, but did observe greatly decreased IFT; we, therefore, speculate that an IFT regulation mechanism plays a role in this shortening and remodelling process. The balance point mechanism of axoneme length control^53^ suggests a model where stochastic dissociation of axoneme components from the distal tip (which occurs during normal axoneme turnover) may give rise to axoneme disassembly when the concentration of axoneme components at the flagellum tip is not maintained by IFT. Assuming this hypothesis implies that the central pair is particularly susceptible to this form of disassembly. In such a case passive diffusion may be the dominant process for the transport of diassembled axoneme components returning to the cell. This would be consistent with the slow disassembly rate here in comparison with 9+2 axoneme shortening in *Chlamydomonas*, which is associated with increased IFT^55^.

Our results also showed that through a differentiation division, one daughter cell can form a 9+0 axoneme directly (Fig. 6). This latter mechanism reveals an important property of basal bodies/centrioles: the pro-basal body in a proliferative promastigote with a long 9+2 flagellum is multipotent. If an individual promastigote enters a normal proliferative cell cycle, the pro-basal body will nucleate a 9+2 flagellum; however, if that promastigote enters an amastigote differentiation cell cycle, then the pro-basal body will nucleate a 9+0 flagellum. Therefore, from its birth until maturation in the next cell cycle, this microtubule triplet-based pro-basal body is multipotent. Definition of the type of flagellum to be elaborated must therefore be acquired during the formation of the doublet microtubule transition zone and docking to the membrane with elaboration of the basal plate and other structures, although the assembled axoneme retains the ability to restructure.

Use of a differentiation division to achieve adaptation of the biochemistry and morphology of one daughter cell to a new life cycle stage is characteristic of trypanosome species, and has been seen in *T. brucei*^56^,^57^ and *Trypanosoma cruzi*^58^. Our evidence for this behaviour in *L. mexicana* shows that similar behaviour occurs among the *Leishmania* spp. In each of these cases the daughter cell that inherits the new basal body and flagellum is the cell that appears immediately adapted to the next life cycle stage, while the fate of the other daughter may be continued proliferation^57^, death^56^,^58^ or more gradual adaptation (*Leishmania*).

The flexibility of axoneme structure and basal bodies demonstrated here have a number of general implications. In a parasitic life cycle, the ability both to remodel an existing flagellum and *de novo* nucleate a different type of flagellum is a useful tactic, as after a cell division both daughter cells can generate the axoneme structure necessary for proliferation in the next life cycle stage. In mammalian biology, the capacity for axoneme remodelling and multipotency of pre-maturation centrioles are important possibilities to consider when dissecting the pathways of cell development that lead to the production of different motile, immotile and sensory axoneme structures in various types of cell and tissue. It will be of considerable interest to understand the regulation of centriole commitment to axonemal type and the process of axonemal remodelling.

## Methods

### Cell culture

*L. mexicana* (WHO strain MNYC/BZ/62/M379) promastigote forms were grown in M199 medium with Earle's salts and L-glutamine (Life Technologies) supplemented with 10% volume/starting volume (v/sv) heat inactivated South American origin FCS (Life Technologies), 5 mM HEPES·NaOH (pH 7.4), 26 mM NaHCO_3_ and 5 μg ml^−1^ haemin, at 28 °C^43^. EPPs were maintained by subculture to maintain culture density between 1 × 10^5^ cells ml^−1^ and 1 × 10^7^ cells ml^−1^ (ref. 43), and grown to stationary phase by growth without subculture for 5 days from 1 × 10^5^ cells ml^−1^ (ref. 33). pH and temperature shift to induce differentiation to axenic amastigotes was triggered by subculture into SM5.5 (Schneider's Drosophila medium (Life Technologies) supplemented with 20% v/sv heat-inactivated FCS and 25 mM MES·HCl (pH 5.5), at 34 °C with 5% CO_2_^37^). *L. mexicana* culture density and cell volumes were measured using a CASY model TT cell counter (Roche Diagnostics) with a 60 μm capillary and exclusion of particles with a pseudodiameter below 2.0 μm as debris. The minimum number of cells assayed for culture density and cell volume measurement was 5,000 per sample.

Murine bone marrow-derived macrophages (BMDMs) were grown in DMEM (high glucose DMEM supplemented with 10% v/sv heat-inactivated FCS). BMDMs were derived from bone marrow monocytes from C57BL/6 mice, differentiated *in vitro* by culture for 8 days at 37 °C with 5% CO_2_ in high-glucose DMEM supplemented with 20% v/sv L929 conditioned DMEM. L929 conditioned DMEM contains macrophage colony-stimulating factor (M-CSF). Carcasses for the extraction of bone marrow cells were supplied by our biomedical services department. The animals were not killed specifically for our use and as they were killed by a Schedule 1 method (as per the Animals (Scientific Procedures) Act 1986) licence authority was not required. Differentiation of monocytes into macrophages was confirmed by immunofluorescence with each of five rat IgG2b antibodies diluted 1:50: Alexa Fluor 647 anti-GR1 (clone RB6-8C5 108420, 19, Biolegend), Alexa Fluor 488 anti-F4/80 (clone BM8 123120, Biolegend), Alexa Fluor 647 isotype control (RTK4530, Biolegend) or Alexa Fluor 488 isotype control (RTK4830, Biolegend). For infection with *L. mexicana*, BMDMs were incubated for 2 days at 34 °C with 5% CO_2_ in DMEM. SPPs were then added at a multiplicity of infection of 3.

### Expression constructs and transgenic cell lines

*Leishmania* axonemal proteins were identified through a literature search for *Chlamydomonas reinhardtii* axoneme proteins that are a part of the main axoneme repeat structures (yielding 63 proteins) and the *L. mexicana* homologues were identified by reciprocal best BLAST with an *E* value cutoff of 10^−5^ using TriTrypDB^59^, yielding 33 putative homologues (Supplementary Data 1) from which panels of cell lines for analysis were selected to include representatives of all major axoneme structures (Table 1). Constitutive expression of eGFP or mCherry (mCh) fluorescent fusion proteins was achieved by fusing the fluorescence gene to the 3′-end of the target gene open reading frame at one of the endogenous loci. Endogenous tagging constructs were derived from the pLEnTv1 *Leishmania* endogenous tagging vector^54^. Primer sequences used and restriction enzymes for vector linearization are listed in Supplementary Table 1. This approach replaced the endogenous target gene 3′-UTR with the *Crithidia fasciculata* phosphoglycerate kinase A (*Cf*PGKA) 3′-UTR in the pLEnT vector, which disrupted any downregulation of expression in the amastigote stage conferred by the endogenous 3′-UTR (such as previously described for PFR2 (ref. 60)). Given gene expression in trypanosomatids is predominantly regulated posttranscriptionally by elements in the 3′-UTR^61^, this method ensures absence of eGFP or mCh signal corresponds to absence of the structure to which that protein binds, rather than downregulation of expression. Expression of eYFP::CEP104 was achieved by generating an endogenous tagging construct using fusion PCR^54^. The primer sequences used were: forward; 5′-AATCGCCACCGTTGTTTGTCG-3′, forward nested; 5′-CAAGTAACGCTCTTGCTTTCC-3′, *bsr* fusion; 5′-GATTCTTCTTGAGACAAAGGCATGAGGAGCGTCCGAATACAATAAG-3′, eYFP fusion; 5′-CGGCATGGACGAGCTGTACAAGATGTTTCACTCCGAGGCGAAG-3′, reverse; 5′-CATCTCATCCAGTGGCACCG-3′ and reverse nested; 5′-GGCGAGTGGGGTATCGACCC-3′ and used pPOTv2 (ref. 54) as a template. Cell lines were generated from cells expressing SMP1::mCh as a flagellar membrane counter label. Expression of the correct fusion protein was confirmed by western blot (Supplementary Fig. 2a), and any detrimental effect of expressing the fusion protein was assessed through growth rate (Supplementary Fig. 2b). Shld1-inducible expression eGFP::RSP11 was achieved by fusion with the ddFKBP domain at the N terminus. Constructs were derived from the pIR1PHLEO-ddFKBP *Leishmania* expression vector^45^ by introducing the RSP11 open reading frame. The primer sequences used were: forward; 5′-TGTACAGGCCTATGGGGCTCATGCGCACA-3′ and reverse; 5′-GTACATTCTAGATTAGTACTCCTCGACGTC-3′. The resulting constructs were linearized by restriction digest with *Swa*I and integrate into the small subunit ribosomal RNA array. *Leishmania* transfections and cloning of cell lines by limiting dilution were done as previously described^54^.

### Immunoblotting

Promastigotes were harvested by centrifugation from culture, washed with PBS then lysed in hot Laemmli buffer. 5 × 10^6^ cell equivalents per sample were subjected to SDS–PAGE on a 12% Bis–Tris gel, and transferred to PVDF membrane. Even sample loading was confirmed by Ponceau staining of the membrane, then the membrane was probed with rabbit anti-GFP antibodies diluted 1:1,000 (A11122, Life Technologies) and horseradish peroxidase-conjugated anti-rabbit IgG diluted 1:5,000 (P0448, DakoCytomation) and detected by chemiluminescence using standard protocols.

### Fluorescence microscopy

*L. mexicana* morphology and cell cycle stage were determined from formaldehyde fixed samples: parasites were harvested by centrifugation at 800*g* for 5 min, washed three times in PBS, then allowed to settle on poly-lysine coated microscope slides for 10 min. Cells were fixed by addition of 2% paraformaldehyde for 5 min, stained with 1 μg ml^−1^ DAPI, then washed with PBS and mounted in glycerol with 10% v/v 50 mM sodium phosphate pH 8.0. Epifluorescence micrographs were captured with a × 40 NA 1.30 objective lens on a DM5500 B microscope (Leica Microsystems) with an Orca charge-coupled device camera (Hamamatsu Photonics) and flagellum length measured in ImageJ^62^.

Cell lines expressing eGFP fusion proteins were imaged live, adhered to glass slides. Parasites were harvested from culture by centrifugation at 800g for 5 min and washed three times in PBS with Hoechst 33342 at 10 μg ml^−1^ in the first wash. The cells were resuspended in 10 μl PBS, placed on a poly-lysine coated microscope slide, then a coverslip was applied and the cells were immediately imaged with a × 100 NA 1.40 plan-apochromat oil immersion objective lens (Leica Microsystems 11506211) on a DM5500 B microscope (Leica Microsystems) with an Orca charge-coupled device (CCD) camera (Hamamatsu Photonics) at the ambient temperature of 25–28 °C.

For live cell time-lapse imaging of infection of BMDMs the BMDM endocytic system was pre-loaded with rhodamine-conjugated dextran, so instances of parasite death could be identified by loss of membrane integrity allowing rhodamine–dextran to enter the parasite cytoplasm. BMDMs from culture at 34 °C were incubated in 35 mm glass-bottom plates at 34 °C with 5% CO_2_ in DMEM supplemented with 250 μg ml^−1^ rhodamine-conjugated dextran (average molecular weight 10,000) for 18 h. The medium was then removed by aspiration and the cells were washed with 2 ml DMEM three times, then infected with *L. mexicana* as described above. Samples were immediately observed on an LSM 510 inverted confocal microscope with a × 40 NA 0.8 objective lens (Zeiss). One set of eGFP fluorescence, rhodamine fluorescence and phase contrast images was captured every 5 min and samples were maintained at 34 °C with 5% CO_2_ using an environmental chamber.

### Electron microscopy

Cells were fixed as previously described^63^,^64^ in suspension in the culture medium with 2.5% v/v glutaraldehyde for 5 min, then harvested by centrifugation and resuspended in 200 mM phosphate buffer (pH 7.0) supplemented with 2.5% v/v glutaraldehyde and 2.0% v/v paraformaldehyde. The fixed cells were pelleted, and transferred to fresh buffered fixative and incubated for 2 h at room temperature. The pellet was then washed in 200 mM phosphate buffer (pH7.0), post-fixed with 1% w/v osmium tetroxide in 100 mM phosphate buffer for 2 h at 4 °C, washed with water then en bloc stained with 2% w/v uranyl acetate for 2 h at 4 °C. The pellet was then washed with water and dehydrated through in an ascending ethanol series and embedded in Agar 100 resin (AGR1031, Agar Scientific). Serial sections of a nominal 90 nm thickness were cut and collected onto formvar-coated slot grids, stained with Reynolds lead citrate for 2 min then washed with water. Electron micrographs were captured on a Tecnai 12 TEM (FEI) and captured with an Ultrascan 1,000 CCD camera (Gatan).

### Image analysis

Averaged views of axoneme structures were generated from digital electron micrographs using ImageJ^62^. For axonemes with a circular cross-section (9+2, 9+0) the elliptical distortion arising from deviation from the perfect transverse section was corrected as previously described^65^, then signal intensity across sections was averaged. Markham rotational averages^66^ were generated using 40° rotation steps around the centre of the axoneme.

For axonemes with a non-circular cross-section or with collapse of the outer doublets (9v) elliptical distortion could not be estimated, and rotational averaging around the centre of the axoneme was nonsensical as the nine-fold axoneme symmetry had been broken. We observed that the doublets did not undergo an axial twist, despite collapse, leaving them oriented at 40° to their neighbours. Signal intensity from doublets was therefore averaged, centred on the A-tubule and assuming 40° rotation from their neighbours.

To determine the distance between the distal tip of an eGFP-labelled axoneme structure and the distal tip of the SMP1::mCh-labelled flagellar membrane we fitted signal intensity to a sigmoidal function. Fluorescence micrographs of endogenous eGFP and mCh fluorescence in flagella were digitally straightened^67^ based on SMP1::mCh signal (Fig. 5b), then the eGFP and mCh fluorescence intensity profile across the flagellar tip was fitted to a modified form of the Rodbard four-parameter logistic curve^68^:


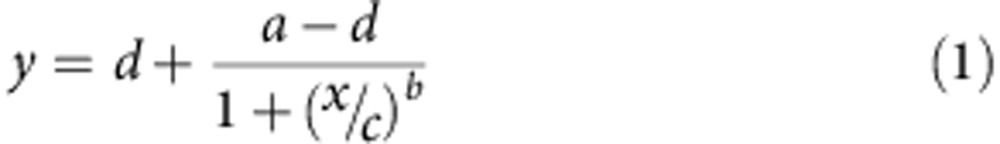


where *a* and *d* are maximum and minimum signal intensity, respectively, *c* is the position of half-maximum (over background) signal intensity and *b* is the sigmoidal steepness factor. Half-maximum signal was taken as the end of the fluorescent structure and was used to determine the distance from the tip of the structure containing eGFP to the membrane that bounds the distal end of the flagellum, marked by mCh. Cases where *c* fell outside the image bounds or where *b* was very shallow were excluded as they give a poor estimate of *c.*

To analyse IFT from IFT52::eGFP native fluorescence videomicrographs captured at 2.74 Hz, the flagella were digitally straightened^67^ based on SMP1::mCh signal. To account for the effect of photobleaching, each frame was normalized to the same signal mean and variance. Non-moving background signal was removed by subtraction of the mean intensity over all frames. To generate the kymographs, signal intensity was summed in the transverse direction, then remapped to give a kymograph with distance along the flagellum in the horizontal direction and time in the vertical direction. IFT particle speed was quantified by automated analysis of the discrete FFT of kymographs. Angle of a track in a kymograph is proportional to the arctangent of particle speed; therefore, signal intensity at different angles in the kymograph was determined by performing a discrete FFT, then summing signal intensity over 1° steps from −90 to 90°. Angle in the FFT was transformed back to velocity based on the kymograph spatial horizontal and temporal vertical scales, and used to plot the corresponding FFT signal intensities. Background signal due to image noise was determined from dummy analysis of digitally straightened lines through the cell body, and was subtracted.

## Additional information

**How to cite this article:** Wheeler, R. J. *et al.* Basal body multipotency and axonemal remodelling are two pathways to a 9+0 flagellum. *Nat. Commun.* 6:8964 doi: 10.1038/ncomms9964 (2015).

## Supplementary Material

Supplementary Figures and Supplementary Table Supplementary Figures 1-3 and Supplementary Table 1

Supplementary Data 1 *Chlamydomonas reinhardtii* axonemal proteins and their human and *Leishmania mexicana* homologs.

Supplementary Movie 1 Time-lapse of flagellum disassembly following promastigote uptake by a macrophage. Example confocal epifluorescence time-lapse videomicrograph of promastigote uptake by a BMDM. BMDMs were pre-incubated with 10,000 Mw rhodamine-conjugated dextran for 18 h prior to infection, then co-incubated with SPPs expressing SMP1::eGFP at a target MOI of 3, at 34°C with 5% CO2 in the environmental chamber of the microscope. Images were captured every 5 min. Instances of parasite death are visible through increased cytoplasmic dextran signal. Stills from this videomicrograph are shown shown in Fig. 2a. Scale bar represents 10 μm.

Supplementary Movie 2 Videomicrographs of IFT in growing and disassembling 9+2 axonemes. Example epifluorescence videomicrograph of IFT in EPPs in M199 (left) and 4 h after transfer to SM5.5 (right), after which the flagella are in a state of disassembly and restructuring to 9+0. Images of cells expressing IFT52::eGFP were captured every 0.365 s. Stills from this videomicrograph are shown shown in Fig. 5a. Scale bar represents 5 μm.

## Figures and Tables

**Table 1:**
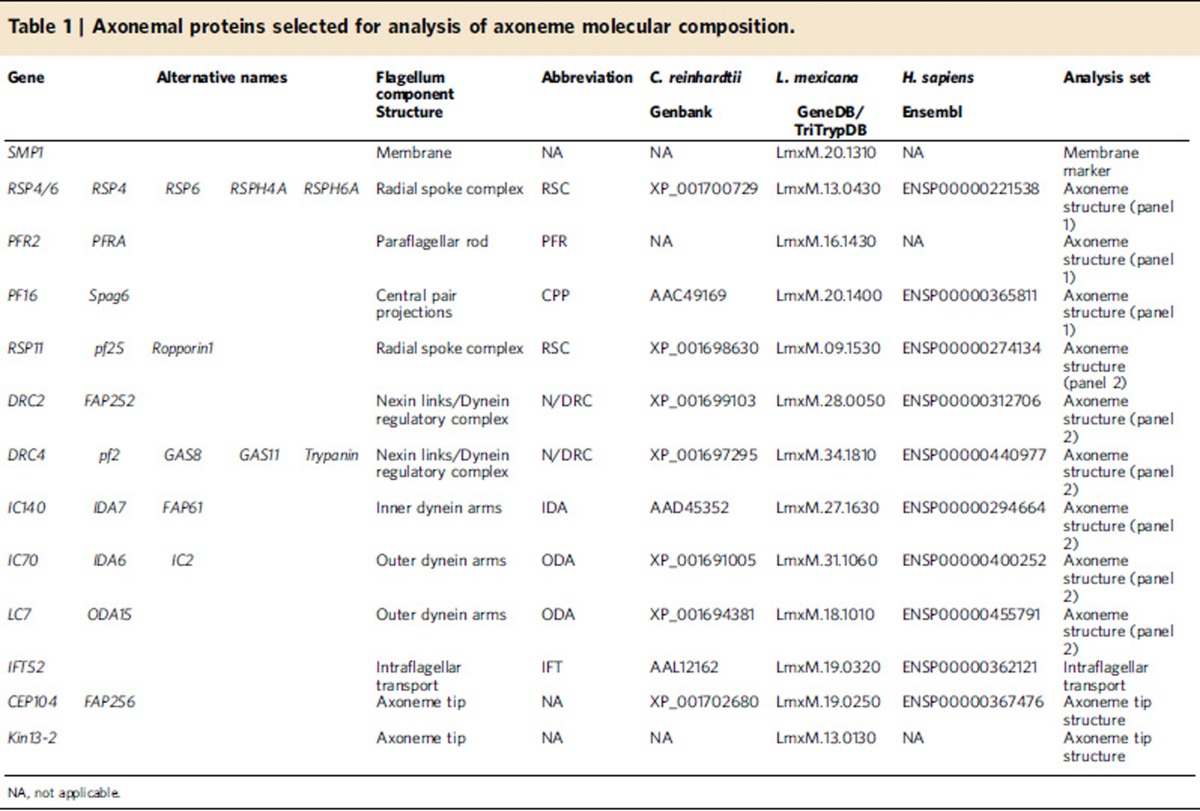
Axonemal proteins selected for analysis of axoneme molecular composition.
