# Supplementary material for: Basal body multipotency and axonemal remodelling are two pathways to a 9+0 flagellum
Source: Nat Commun. 2015 Dec 15;6:8964. doi: 10.1038/ncomms9964 (PMC4682162; doi:10.1038/ncomms9964)
Supplement: Supplementary Figures and Supplementary Table — Supplementary Figures 1-3 and Supplementary Table 1 [file ncomms9964-s1.pdf]

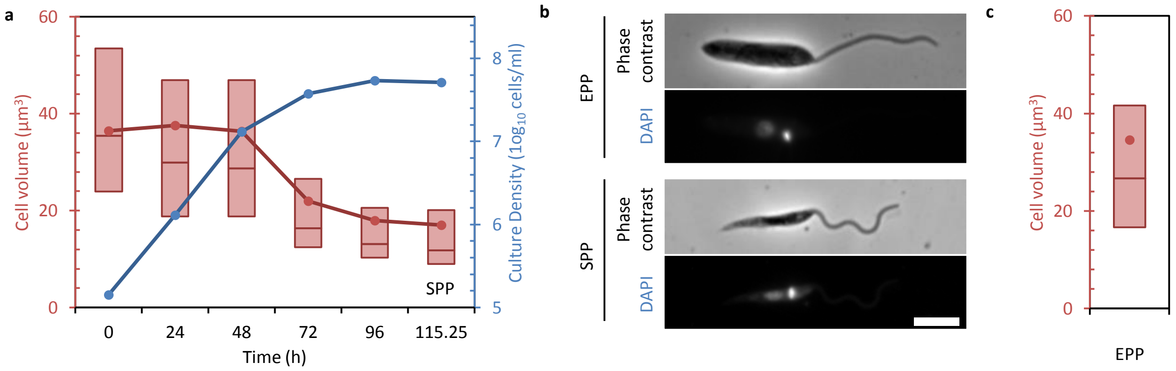

Supplementary Figure 1: Growth to stationary phase generates promastigotes morphologically similar to metacyclic promastigotes. (**a**) Culture density and mean cell volume of a representative promastigote culture during growth to stationary phase in M199 at pH7.4, 28°C, showing a significant (KS test, $p<0.001$) reduction of cell volume on reaching stationary phase. Points indicate the mean and boxes represent median, upper and lower quartiles. Metacyclic promastigotes have a maximum length and width of 8.0 and 1.5 µm respectively, corresponding to a volume of <10 μm^3 assuming a prolate spheroid shape. (**a**) representative of two independent experiments. (**b**) Phase contrast and DAPI epifluorescence micrographs of a representative 1K1N EPP and SPP. Scale bar represents 5 µm. (**c**) Cell volume, presented as in (**a**), of EPPs maintained by daily subculture to 1×10^6 cells/ml. These cells retained a large cell volume. Pooled data from volume measurement on 3 successive days, (**c**) representative of two independent experiments.

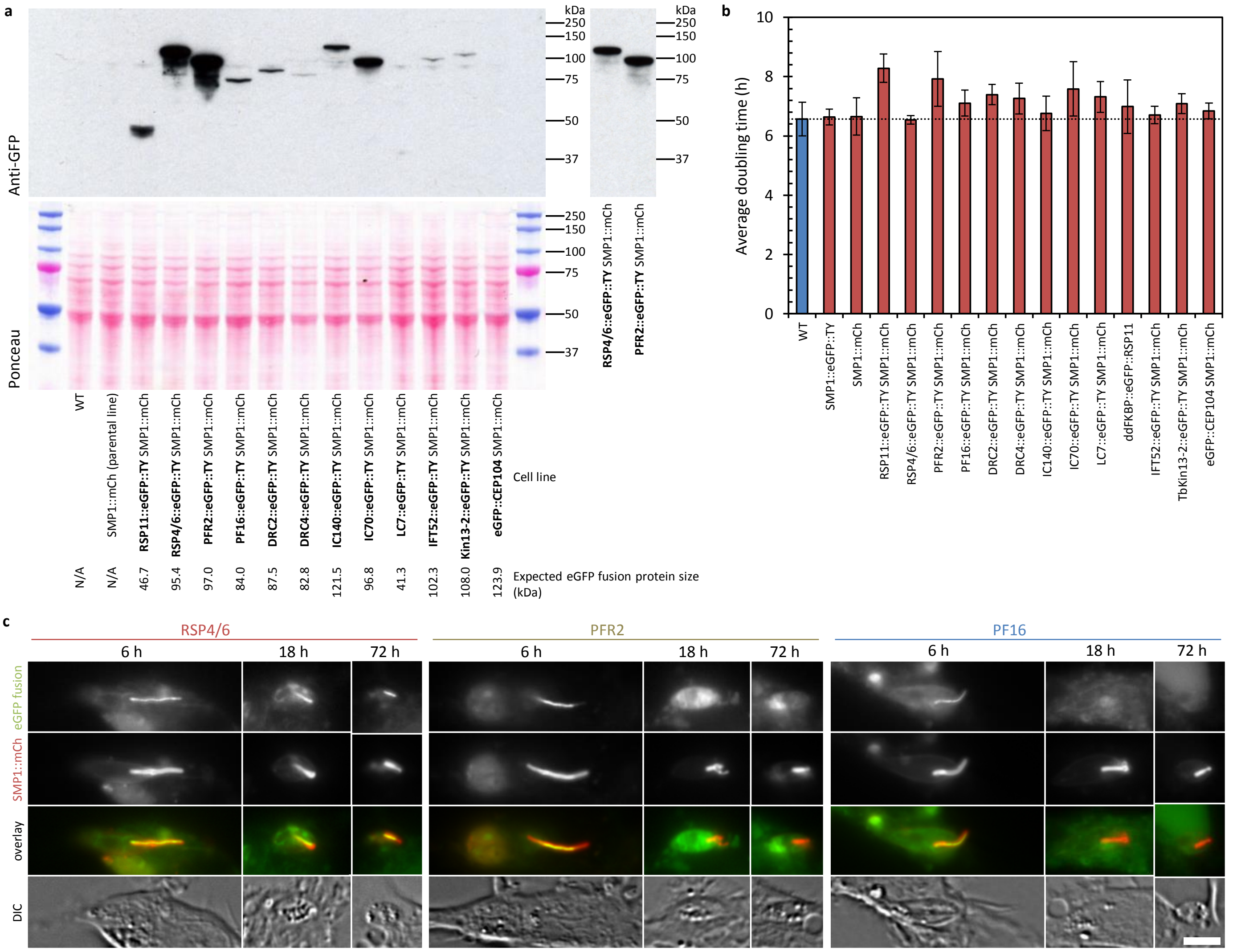

Supplementary Figure 2: A set of *Leishmania* cell lines for analysis of axoneme composition. (**a**) Expression of the expected eGFP fusion protein was confirmed by anti-GFP Western blot of whole cell lysate from cells in M199 for each of our set of cell lines, representative of a single experiment. A shorter exposure of the RSP4/6 and PFR2 section of the membrane is shown in the top right. An eGFP fusion protein near the expected size was detected for all cell lines, except eGFP::CEP104 where no fusion protein could be detected. Despite sharing the same 3' UTR, expression level was highly variable. (**b**) Average doubling time of each of our panel of cell lines was determined as EPPs over three 24 h periods. Error bars are the standard deviation. No cell line showed a large deviation from the wild type growth rate. (**c**) Summary of native fluorescence micrographs of parasites 6, 18 and 72 h after update of SPPs by a macrophage from panel 1 of cell lines expressing eGFP fusions of known axonemal proteins (Table 1). Representative images from two macrophage infections. Axonemal protein localisation was extremely similar to that seen during axenic differentiation of EPPs (Fig. 1g,h). Scale bar in (**c**) represents 5 µm.

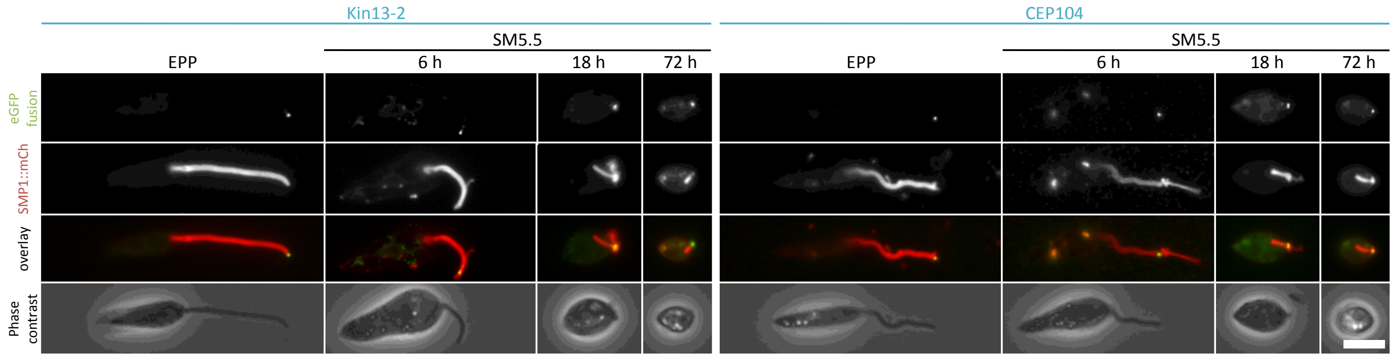

Supplementary Figure 3: 9+2 axoneme shortening and restructuring to 9v does not alter localisation of tip proteins. Example epifluorescence micrographs of native fluorescence in live cells expressing eGFP fusions of axonemal tip proteins. Kin13-2::eGFP or eGFP::CEP104 are present at the flagellum tip in EPPs and cells 6, 18 and 72 h after transfer of EPPs to SM5.5. Representative of a single experiment. Scale bar represents 5 µm.

Supplementary Table 1. Primer sequences for tagging of axonemal proteins with pLENTv1.

Gene		ORF fragment		UTR fragment		Linearisation enzyme
**Name**	**GeneDB/TriTrypDB**	**Forward primer**	**Reverse primer**	**Forward primer**	**Reverse primer**	
SMP1	LmxM.20.1310	ATGTACAGATCTATGGGCTGCGGTGCTTCG	GTACATACTAGTCTTTTCCTTCTCCCCCTG	ATGTACAAGCTTGAGCGAACAAACCAATGA	GTACATAGATCTTCGCCCCACCACCACCAC	*Bgl*II
RSP4/6	LmxM.13.0430	ATGTACAGATCTTCTACGGGGTCGTCGACA	GTACATACTAGTGCTGTCCTCCTCTTCCTC	ATGTACAAGCTTAAAGAGGTTCCGCGGGTG	GTACATAGATCTTCCCAGAGGAAAACCGTC	*Bgl*II
PFR2	LmxM.16.1430	ATGTACAGATCTAGCTGCGGCTGCAGGTGC	GTACATACTAGTCTCGGTGATCTGTTGCAC	ATGTACAAGCTTGGGCGGCGCCGCTGGAAA	GTACATAGATCTTCCACGAGACCAGCAAGG	*Bgl*II
PF16	LmxM.20.1400	ATGTACAGATCTTGGAGAAAGAGCCCGAAG	GTACATACTAGTGTGCTGCTGCACGTGGTA	ATGTACAAGCTTGCGGGGGAGGAGGCGTCG	GTACATAGATCTCCAAGCCTCTTCGCGAGA	*Bgl*II
RSP11	LmxM.09.1530	ATGTACAGATCTATGAACGCCATGTACTCT	GTACATACTAGTATAACCCATACCCTCGAA	ATGTACAAGCTTGCGCTCCTGCGCAGAAGT	GTACATAGATCTGCTTCGGAGGCATCTTCG	*Bgl*II
DRC2	LmxM.28.0050	ATGTACAGATCTGTCTTCTGCGGCTAGTGG	GTACATACTAGTGCGATTGATGGCTTGACG	ATGTACAAGCTTGCCACTAGTGAGAGTCTT	GTACATAGATCTGGACATCAGAGAAACAGC	*Bgl*II
DRC4	LmxM.34.1810	ATGTACAGATCTACTACCTGGCGGACAAGG	GTACATACTAGTTGCACGAATCGACGCTGC	ATGTACAAGCTTAAAAGAGGAAAAAGAAGC	GTACATAGATCTTAGCATACATACGCCTCT	*Bgl*II
IC140	LmxM.27.1630	ATGTACAGATCTCCATCACATCTATCGCCT	GTACATACTAGTGGCGGCCGACTCCAACTC	ATGTACAAGCTTAGTTGCCTGGAGCAGCCG	GTACATAGATCTTTTTACTACATCACACAT	*Pac*I
IC70	LmxM.31.1060	ATGTACAGATCTTCTTCACGACTCGCATGG	GTACATACTAGTGTCGTAGAGATCTATGCC	ATGTACAAGCTTGTCTGCTGGTATTGCTCC	GTACATAGATCTCAACGCACAGAGCCATGT	*Pac*I
LC7	LmxM.18.1010	ATGTACAGATCTATGGCTGCCGCCGGCTCC	GTACATACTAGTCTTCTTAGCCTCTACCTT	ATGTACAAGCTTAGTGGGTGCGTGTGTCCG	GTACATAGATCTAAAACCTCAGCTCTTCGC	*Bgl*II
IFT52	LmxM.19.0320	ATGTACAGATCTTCGACTACGTGTTCCGCC	GTACATACTAGTCAGCTCTTCTAGCGTATA	ATGTACAAGCTTGTTCGGAAGCGGCGGTTG	GTACATAGATCTCTTGGGGAGGAAGCAAGA	*Bgl*II
Kin13-2	LmxM.13.0130	ATGTACAGATCTCGACAGCCCAGACGGCAG	GTACATACTAGTCTTGTCCAGACGCTCCAT	ATGTACAAGCTTAGTCGCGGCAGGGCGAGT	GTACATAGATCTAGACACACACACACAACG	*Bgl*II
